# Is Antigenic Sin Always “Original?” Re-examining the Evidence Regarding Circulation of a Human H1 Influenza Virus Immediately Prior to the 1918 Spanish Flu

**DOI:** 10.1371/journal.ppat.1004615

**Published:** 2015-03-05

**Authors:** Alain Gagnon, J. Enrique Acosta, Joaquin Madrenas, Matthew S. Miller

**Affiliations:** 1 Département de Démographie, Université de Montréal, Montreal, Quebec, Canada; 2 Department of Microbiology and Immunology and Microbiome and Disease Tolerance Centre, McGill University, Montreal, Quebec, Canada; 3 Department of Biochemistry and Biomedical Sciences, Institute for Infectious Diseases Research, McMaster Immunology Research Centre, McMaster University, Hamilton, Ontario, Canada; The Fox Chase Cancer Center, UNITED STATES

What makes the 1918 Spanish influenza pandemic stand out from all the others is its well-known W-shaped mortality signature, which was caused by unusually high mortality among adults aged 20 to 40 [1]. Much debate remains as to the exact reason for this atypical pattern [2]. A contribution by Worobey et al. [3] published recently in the *Proceedings of the National Academy of Sciences* (PNAS) is no doubt adding important information to this debate.

In agreement with previous work [4–7], Worobey et al. propose that the very high mortality experienced by young adults during the 1918 H1N1 virus pandemic was primarily due to their childhood exposure to the heterosubtypic H3-like virus that is thought to have caused the earlier 1889–1890 Russian flu pandemic [3]. As is generally accepted, the authors also presume that older adults had immunological cross-protection from earlier exposures to a putative H1-like virus, which circulated prior the 1890 pandemic. As for the lower mortality of children and adolescents, however, a new and compelling hypothesis is put forward: this pattern may be attributed to the appearance of a new H1 influenza variant in the early 1900s, which would have provided protection in 1918 for individuals born at the turn of the century, presumably exposed early in life (or “primed”) to this new variant. They propose that this H1N8 virus arose from reassortment between an H1 lineage virus and an avian influenza virus sometime between 1901 and 1907, replacing the H3N8 virus of the 1889–1890 pandemic. This phylogenetic reconstitution appears to be supported by (often forgotten) seroarcheological and mortality evidence.

## Did H1 Really Replace H3 in the Early 1900s?

Although the phylogenetic analysis presented by Worobey et al. is quite appealing, the results gathered from the seroarcheological literature that they abundantly cite do not always support it [3]. Most notably, it is not immediately clear that all seroarcheology and mortality data point to the swift replacement of the 1890 H3 virus by an H1 variant at the turn of the 20th century. Furthermore, any seroarcheological data gathered to measure responses to viruses for which isolates are not available must be interpreted cautiously.

**Fig 1:**
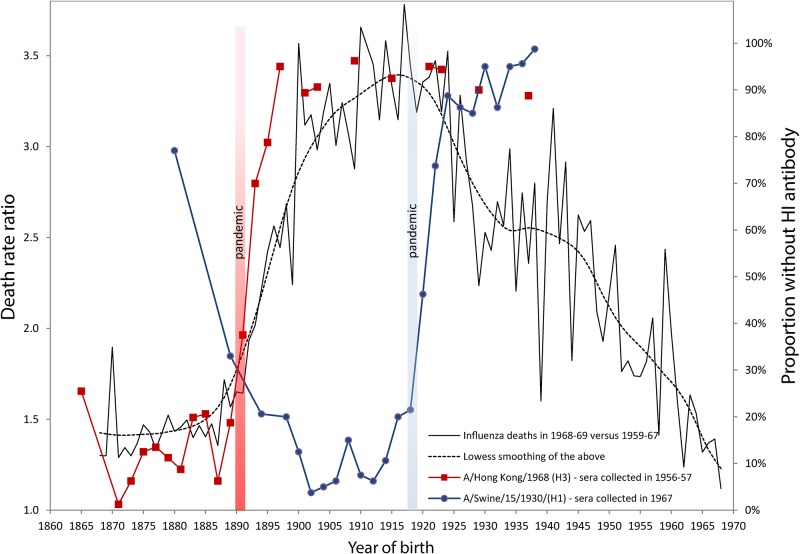
Death rate ratios from pneumonia and influenza (P&I) during the 1968–1969 pandemic and percent without antibody titers against H1 and H3 influenza viruses. Serological data on H1 and H3 viruses were adapted from Masurel [8,9,25] and Doodle et al. [10]. Years of birth were deduced from data originally presented by age by Masurel (HI > 9–19) [8,9,25]. We used life tables from the Netherland Bureau of Statistics, available in the Human Life-Table Database [26], in order to estimate average ages (and years of birth) for donor sera grouped in age bins larger than five years. The death rate ratio by age (or by birth cohort) in 1968 was estimated by dividing the P&I death rate calculated for the December 1968 to January 1969 pandemic flu season [27] with the average P&I death rates of the same seasons from 1959–1960 to 1967–1968. These ratios represent the increase of mortality due to the 1968 pandemic (a ratio of 1.5 means a mortality increment of 50% in comparison with the previous ten-year’s average). Monthly P&I death counts were taken from the National Center for Health Statistics, available on the National Bureau of Economic Research website [28], while the populations exposed to risk were interpolated from the Human Mortality Database [29]. In order to account for secular mortality improvements, we detrended the time series from 1959 to 1968 with quadratic regressions of mortality rates based on the 20 epidemic seasons from 1959 to 1978, excluding the 1968–1969 pandemic season.

Using old serological studies from Masurel [8,9] adapted here in Fig. 1, other investigators such as Dowdle [10] deduced earlier that about half of those born a few years after the 1890 pandemic, i.e., in 1893, were “primed” to the H3 virus that caused that pandemic while the other half would have been primed to H1N1 during the 1918 pandemic. According to Worobey et al., this is highly unlikely because it would mean that many members of this cohort fully escaped all influenza virus infections for a period of about 25 years [3]. However, Dowdle’s explanation for the H1 seroacheological data is only problematic if we adhere to a historical interpretation of “original antigenic sin” by assuming that the highest antibody titres in a birth cohort systematically reflect the antigens of the earliest childhood exposure to influenza virus and that, as a consequence, seroarcheological studies invariably reveal the identity of the first strain of influenza virus to which each cohort were exposed.

This assumption is not always sound [2], as can be readily observed in Fig. 1, which shows that cohorts born between 1863 and 1890 all had a high percentage of individuals with detectable antibodies against the A/Hong Kong/68 (H3) strain. About 80% to 90% of sera collected in 1956–1957 for these cohorts contained HI antibodies against this strain (red line with squares). The maximum proportion, virtually 100%, occurs for those born in 1871, i.e., almost 20 years before the 1890 pandemic. If it is true that the antibody signature resulting from the first influenza virus infection during one’s lifetime is hierarchically programmed into the immunological repertoire of a cohort, then we are forced to suppose that individuals born in the 1860s or 1870s escaped exposure and infection to the putative H1-variant that circulated prior to the 1890 pandemic for a very long period. This is, indeed, highly unlikely. Similarly, the proportion of individuals with antibodies against A/Swine/15/1930/(H1) is over 90%–95% in Fig. 1 for cohorts born long before the appearance of the strain in 1918 (blue line with circles). This trend was also observed recently in sera collected just prior to the 2009 H1N1 pandemic. Those born during the first two decades of the 20th century, presumably exposed early in life to the 1918 Spanish Flu pandemic, had high neutralizing antibody titers against the 2009 H1N1 virus. These titers dropped sharply for those born after 1918, who were exposed to the antigenically distinct “human” H1N1 viruses that circulated from the 1920s to the 1950s [11]. Other independent studies have also reported similar sudden drops in antibody titers against the H3 pandemic and the H1 swine strains [12,13].

## For Enhanced Protection in the Future, Get Your “Pandemic Flu Shot” Now

The sudden increase of the percentage of people born after 1890 with no detectable antibody titers to the H3 virus shown in Fig. 1 would seem to be too steep and too tightly associated with the 1890 pandemic to be interpreted as a sign that H3 was replaced by H1 in 1900 as the first exposure strain for those born in the 1890s. What these seroarcheological observations might instead suggest is that exposure to a pandemic strain any time before ~20 years of age can “reprogram” the antibody repertoire by inducing the most robust antibody titers against the pandemic virus, at the expense of any previously encountered non-pandemic strain, whether from the same subtype or not. This would evidently be sufficient to provide increased protection in a subsequent outbreak of the same subtype [10]. The degree of protection would then rapidly fall for cohorts born right after the pandemic, as the virus drifts and becomes seasonal.

Our own analyses of mortality from P&I during the 1968 pandemic are consistent with this “immunological refocusing” scenario, occurring for those exposed to a pandemic strain at a sufficiently young age. We calculated and added to Fig. 1 the death rates ratios of P&I mortality during the 1968–1969 Hong Kong pandemic relative to the average P&I mortality during the years 1959–1967 in the United States. It is clear that people born between 1878 and 1890 had the lowest mortality increments during that pandemic. The relative risk of mortality sharply increased for those who were born immediately after the 1890 pandemic, in concert with the increase in the percentage of individuals who had no detectable HI antibodies to H3N2.

Individuals born up to 20–25 years before the 1890 pandemic all had about the same protection against the H3N2 virus of the 1968 Hong Kong pandemic. While persons born around 1870 might very well have first “committed” to an H1-like variant early in life, most of the sera collected years later (in 1956–1957) from these people contained large amounts of antibodies to the H3 virus, to which they were exposed in the early 1890s (20 years after their birth) and which offered them substantial protection about 80 years later, during the 1968 outbreak. These results on mortality risk ratios are consistent with a study from Simonsen et al. [14] who found that the risk of influenza-related mortality among the elderly aged 75+ did not increase during the 1968 pandemic relative to the 1975–1976 and 1980–1981 influenza seasons, which were relatively severe. Intriguingly, death rates ratios in 1968 as summarized by the lowess smoothing in Fig. 1 attain a maximum for the cohorts born just before the 1918 pandemic, as if being born during pandemic years carried an increased risk of mortality to a subsequent pandemic caused by a heterosubtypic influenza virus.

## Refining the “Original Antigenic Sin” Doctrine

Implicit in Worobey et al.’s scenario is the notion that the first antigenic variant encountered during childhood systematically conditions immunity for the rest of someone’s life [3]. This phenomenon, referred to as “original antigenic sin” (OAS), has been described since the early 1950s, when sequential exposures to drifting variants of the H1N1 subtype seemed to induce more neutralizing antibody titers against the childhood variant than against the contemporary circulating strain of the same subtype [15].

Recent studies have challenged the historical mechanistic implications of the OAS model [16–18], and have proposed the term “antigenic seniority” as a more apt description for the hierarchical nature of antibody responses to previously encountered influenza virus strains. Indeed, the term “OAS” itself has often been used in the literature to describe phenomena related to immunological memory that are not directly linked to the hierarchical responses to influenza virus (as in [14]). To avoid confusion, other research has also proposed a more general conception of antigenic imprinting [19] that would cover all instances of ‘‘commitment” to the strain of first exposure, including sequences involving heterosubtypic pandemic strains like those that caused the 1890 and the 1918 pandemics [5].

It was recently found and confirmed in various locations that mortality during the 1918 pandemic peaked at the exact age of 28 [5,6,20,21] (but see [7,22]), which corresponds to a birth year of 1890 (i.e., during the Russian flu pandemic). To account for this striking regularity, it has been speculated that the development of immunological memory to an influenza virus strain early in life may lead to a dysregulated immune response when encountering a novel and highly antigenically dissimilar strain later in life [5]. For example, encounter with the 1889–1890 H3 virus very early in life would have resulted in robust cytotoxic T cell memory, which, without the complement of cross-protective antibodies between H3 and H1, may have caused immunopathology when recalled upon infection during the 1918 pandemic, resulting in increased risks of death. Those born later in the 1890 decade were primed to progressively drifted and less virulent strains of the H3 virus. This would have decreased the magnitude of the cytotoxic T cell memory response and, thus, lowered the potential for immunopathology in 1918. Similarly, in this study, mortality increments during the 1968 pandemic peaked for those born around one year before the 1918 pandemic, and decreased for those born in the following years. It is worth noting that despite the fact that no new H3-like variant appeared between 1918 and 1968, death rates ratios in 1968 dropped for those born after 1918. In this case, there was no need for circulation of a novel H3 virus to account for this drop, as Worobey et al.’s scenario for the 1918 pandemic would imply if it was applied to the 1968 case [3].

The specific scenarios that result in OAS-like antibody responses are complex, and likely require further refinement (i.e., distinguishing sequences of infections from seasonal virus to pandemic virus, and vice-versa, or sequences involving heterosubtypic pandemic strains). Unfortunately, the disproportionate protection against heterosubtypic infection afforded by T cells in mice makes recapitulating the effects of these exposure scenarios difficult in the mouse model [23,24], and the tools required to study the contribution of specific cell types in ferrets remain lacking. We believe that a detailed understanding of these scenarios will be essential if we ever hope to understand how previous exposures to influenza virus are likely to shape the outcome of future pandemics. More importantly, this knowledge may be critical in the design and implementation of immunization campaigns that are “personalized” with regard to age and exposure history.
